# Supplementary material for: The association between socioeconomic disadvantage and children’s working memory abilities: A systematic review and meta-analysis
Source: PLoS One. 2021 Dec 2;16(12):e0260788. doi: 10.1371/journal.pone.0260788 (PMC8639069; doi:10.1371/journal.pone.0260788)
Supplement: S1 File — (DOCX) [file pone.0260788.s001.docx]

# Supplementary online materials

## 1. Search strategy (Embase)

Database: Embase <1974 to 2019 May 10>

Search Strategy:

1 demography/ (198194)

2 environmental planning/ (10001)

3 marriage/ (53343)

4 divorce/ (7020)

5 cohabitation/ (797)

6 widow/ (2247)

7 exp "single (marital status)"/ (6150)

8 neighbo?rhood*.mp. (32075)

9 residential environment*.mp. (764)

10 rural*.mp. (175407)

11 inner?city.mp. (154)

12 housing instability.mp. (283)

13 housing insecurity.mp. (94)

14 housing strain.mp. (4)

15 housing security.mp. (31)

16 mortgage problems.mp. (1)

17 foreclosure.mp. (224)

18 eviction*.mp. (968)

19 housing loss.mp. (21)

20 home repossession*.mp. (3)

21 home ownership.mp. (446)

22 (repossess* adj3 hous*).mp. (5)

23 (repossess* adj3 propert*).mp. (1)

24 mortgage delinquency.mp. (6)

25 mortgage arrears.mp. (1)

26 mortgage debt*.mp. (6)

27 overcrowding.mp. (3382)

28 (living adj1 (outside or inside or near* or adjacent)).mp. (3894)

29 (household adj2 size).mp. (1324)

30 (marital status or marraige status).mp. (27781)

31 (widow* or cohabit* or divorce* or single parent* or live* alone).mp. (29405)

32 or/1-31 (479876)

33 exp cultural deprivation/ (548)

34 cultural factor/ (58667)

35 cultural anthropology/ (51094)

36 cultural diversity/ (1234)

37 exp migrant/ (33046)

38 minority group/ (13877)

39 Minority health/ (705)

40 Prejudice/ (2467)

41 exp Social Discrimination/ (15797)

42 exp Race Relation/ (5475)

43 exp Ethnic Group/ (127705)

44 exp ancestry group/ (272852)

45 exp refugee/ (11699)

46 minorit*.mp. (90473)

47 migration background.mp. (581)

48 racial.mp. (72070)

49 racism.mp. (6702)

50 ethnology.mp. (70258)

51 race.mp. (183865)

52 ethnic*.mp. (260455)

53 non?English.mp. (32)

54 language other than.mp. (478)

55 latino*.mp. (15454)

56 latina*.mp. (4760)

57 hispanic*.mp. (82326)

58 whites.mp. (32702)

59 caucasian*.mp. (150831)

60 non?white.mp. (3119)

61 Torres Strait Islander.mp. (1722)

62 aboriginal.mp. (10037)

63 native american.mp. (4756)

64 inuit.mp. (2187)

65 eskimo.mp. (2479)

66 first nation*.mp. (5586)

67 indigenous.mp. (37432)

68 english as a second language.mp. (1138)

69 foreign language.mp. (1059)

70 or/33-69 (866135)

71 exp employment status/ (29787)

72 job characteristics/ (620)

73 occupations.mp. (14022)

74 unemployment.mp. (21779)

75 or/71-74 (47648)

76 exp Gender Identity/ (15903)

77 Women's Health/ (26268)

78 sex difference/ (345642)

79 (sex disparit* or sex difference?).mp. (355742)

80 gender identity.mp. (17270)

81 sex role.mp. (5714)

82 wom#n* role?.mp. (578)

83 m#n* role?.mp. (11172)

84 gender* role?.mp. (3217)

85 servicewomen.mp. (100)

86 or/76-85 (411158)

87 exp Educational status/ (68421)

88 Schooling.mp. (8965)

89 educational status.mp. (68348)

90 (education* adj2 level?).mp. (59929)

91 ((higher or better or worse or less) adj educated).mp. (6094)

92 ((higher or better or worse or less) adj level? of education).mp. (2493)

93 or/87-92 (126584)

94 Religion/ (64221)

95 religi*.mp. (82104)

96 or/94-95 (82104)

97 "social determinants of health"/ (5401)

98 social aspect/ (74211)

99 working poor/ (41)

100 exp social hierarchy/ (6125)

101 socioeconomics/ (131773)

102 disparit*.mp. (92242)

103 inequalit*.mp. (34016)

104 inequit*.mp. (9798)

105 equity.mp. (16383)

106 deprivation.mp. (100919)

107 gini.mp. (1288)

108 concentration index.mp. (1734)

109 Social Welfare/ (17373)

110 social class/ (29351)

111 Poverty/ (42260)

112 social status/ (81083)

113 social background/ (550)

114 Social class*.mp. (34366)

115 social determinants.mp. (9553)

116 social status.mp. (84396)

117 social position.mp. (1077)

118 social background.mp. (1720)

119 social circumstance*.mp. (1532)

120 socio-economic.mp. (37754)

121 socioeconomic.mp. (99231)

122 sociodemographic.mp. (51530)

123 socio-demographic.mp. (31733)

124 SES.mp. (23628)

125 disadvantaged.mp. (13525)

126 impoverished.mp. (3709)

127 poverty.mp. (52323)

128 economic level.mp. (1297)

129 assets index.mp. (22)

130 income*.mp. (162976)

131 or/97-130 (774668)

132 exp social isolation/ (20730)

133 social capital/ (2277)

134 Social Stigma/ (6879)

135 Social Support/ (84008)

136 social environment/ (31486)

137 trust/ (17965)

138 exp social exclusion/ (1382)

139 anomie/ (62)

140 social participation/ (5201)

141 social exclusion.mp. (2692)

142 (social adj (capital or cohes* or organis* or organiz*)).mp. (7258)

143 (community adj3 (cohes* or participa*)).mp. (21103)

144 ((neighbourhood or neighborhood) adj cohes*).mp. (148)

145 social relationships.mp. (6418)

146 social network*.mp. (23409)

147 collective efficacy.mp. (418)

148 civil society.mp. (1963)

149 informal social control.mp. (107)

150 neighbo*rhood disorder.mp. (208)

151 social disorgani?ation.mp. (229)

152 anomie.mp. (277)

153 social support.mp. (94211)

154 social participation.mp. (6843)

155 trust.mp. (52112)

156 emotional support.mp. (7362)

157 psychosocial support.mp. (4701)

158 community capital.mp. (13)

159 neighbo*rhood cohesion.mp. (144)

160 social influence.mp. (2020)

161 (soci*context* or soci*-context*).mp. (11584)

162 or/132-161 (265827)

163 health disparity/ (16941)

164 health equity/ (1984)

165 health care access/ (56085)

166 health*care disparit*.mp. (1177)

167 health care disparit*.mp. (14091)

168 health status disparit*.mp. (423)

169 health disparit*.mp. (24421)

170 health inequalit*.mp. (6053)

171 health inequit*.mp. (1818)

172 medically underserved.mp. (2388)

173 or/163-172 (97251)

174 32 or 70 or 75 or 86 or 93 or 96 or 131 or 162 or 173 (2395489)

175 Short Term Memory/ (19103)

176 working memory/ (36513)

177 Executive Function/ (34355)

178 ("working memory" or "executive function*" or "short?term memory").mp. (86194)

179 (Child* or infant or school child* or adolescen* or preschool* or pre-school* or boy* or girl* or young people or teenager* or teen* or youth*).mp. [mp=title, abstract, heading word, drug trade name, original title, device manufacturer, drug manufacturer, device trade name, keyword, floating subheading word, candidate term word] (3482754)

180 Child/ (1577966)

181 179 or 180 (3482754)

182 175 or 176 or 177 or 178 (98374)

183 174 and 181 and 182 (3932)

*Note: search was repeated and updated for records published between 10^th^ May 2019 – 3^rd^ June 2021.*
