# Supplementary material for: The association between socioeconomic disadvantage and children’s working memory abilities: A systematic review and meta-analysis
Source: PLoS One. 2021 Dec 2;16(12):e0260788. doi: 10.1371/journal.pone.0260788 (PMC8639069; doi:10.1371/journal.pone.0260788)
Supplement: S2 File — (DOCX) [file pone.0260788.s002.docx]

# Supplementary online materials

## 2. Table of study characteristics of all included studies

| **Study details** | | | **Participant details** | | | | **Exposure measure** | **Outcome measure** | | **Risk of bias** |
| --- | --- | --- | --- | --- | --- | --- | --- | --- | --- | --- |
| **Author name** | **Study location** | | **Total n *(% male)*** | **Age range *(M±SD)*** | | **Ethnicity/race/**  **language** | **Socioeconomic Position Indicator**  ***(n in each group)*** | **Working Memory task *(reference)*** | **Effect size** |  |
| **Studies included in meta-analyses** | | | | | | | | | | |
| Albert et al., 2020** | Southeast USA | | 203 (50%) | 8-13 years  (8.6±0.6)  (9.6±0.6)  (10.6±0.7)  (13.2±0.4) | 32.5% European American, 33.5% African American, 34% Latin American | | (i) Parental educational attainment  (ii) Family income at age 9 and 10 | 1. Verbal working memory (Thompson-Schill et al., 2002) 2. Spatial working memory – Corsi task (Chein and Morrison, 2010) | Converted from Pearson’s r | LOW |
| Alloway et al. 2014 | England | | 264 (48%) | 51 – 68 months  (NR) | 100% British | | A Classification of Residential Neighbourhoods (ACORN)  *(low SEP n = 123, high SEP n = 141)* | 1. BDR  2. Counting recall  3. Word recall  (WMTBC, 2001) | Cohen’s d | HIGH |
| Arán-Filippetti, 2013 | Santa Fe, Argentina | | 248  (NR) | 8 – 12 years  (NR) | 100% Spanish speaking | | Socioeconomic coefficient of schools  *(low SEP n = 124, high SEP n = 124)* | 1. FDR  2. BDR  3. Letter number sequencing  (WISC-IV) | Cohen’s d | LOW |
| Babayiǧit, 2014 | England | | 168 (26%) | NR  (115.38±3.57 months) | 45% White British, 1 % White-Irish, 1% White Traveller Irish, 1 White Gypsy Roma, 2% Black-Caribbean, 15% Black-African, 11% Asian-Pakistani, 7% Asian-Bangladeshi, 4% Black-any other, 3% Asian-any other, 1% Chinese, and 9% White-any other background | | Free School Meals  *(low SEP n = 53, high SEP n = 115)* | 1. Listening recall  (WMTB-C, 2001) | Converted from Pearson’s r | HIGH |
| Bowey, 1995 | Brisbane, Australia | | 48  (NR) | 5  (65.11±4.44 months) | 100% Native English speakers | | Australian Standard Classification of Occupations (ASCO)  *(low SEP n = 25, high SEP n = 23)* | 1. Digit span (WISC)  2. Nonword repetition | Cohen’s d | HIGH |
| Carlson and Meltzoff, 2008 | Unknown | | 50  (52%) | 58 – 83 months  (72.3±5.33) | 76% Monolingual English speakers, 24% bilingual Spanish/English speakers | | (i) Maternal education  (ii) Paternal education  (iii) Annual family income | 1. Visually cued recall | Converted from Pearson’s r | LOW |
| Catale et al, 2012 | Belgium | | 64  (30%) | 6 – 7 years and  10 – 11 years  (NR) | 100% European native French speakers | | Parental education  *(low SEP n = 32, high SEP n = 32)* | 1. Digit span  2. WM task (Test Battery for Attentional Performance, Zimmermann and Fimm, 1994) | Cohen’s d | LOW |
| Chung et al., 2017 | Hong Kong | | 199  (50%) | 44 – 67 months (58.25±3.76) | 100% Chinese | | Parental education, parental occupation and income-to-needs ratio  [Composite]  *(low SEP n = 97, high SEP n = 102)* | 1. FDR  2. BDR  (WISC-III) | Cohen’s d | LOW |
| Corso et al. 2016 | Southern Brazil | | 110  (51%) | 9 – 12 years  (135.85±12.46 months) | 100% Brazilian | | Brazillian Associação Brasileira de Empresas de Pesquisa (ABEP) (possession of goods, purchase of services, parental education)  [Composite] | 1. Pseudo word span  2. FDR  3. BDR  (Child Brief Neuropsychological Assessment Battery, 2011) | Converted from Pearson’s r | LOW |
|  |  | |  |  |  | |  |  |  |  |
| Deer et al. 2020** | UK  Avon Longitudinal Study of Parents and Children (ALSPAC) | | Approximately 7006 (53%) | 0-5 years (NR), 8 years (NR) and  10 years (NR) | 96% White | | (i) Family income at ages 0-5  (ii) Parental education at age 8 | 1. Counting Span task at age 10 (Case, Kurland, & Goldberg, 1982) | Converted from Pearson’s r | LOW |
| Engel de Abreu et al. 2014 | Sao Paulo City, Brazil | | 355  (49%) | 6 – 8 years  (89.11±7.84 months) | 45% White, 11% Afro-Brazilian, 42% multiracial | | Public and private schools  *(low SEP n = 182, high SEP n = 173)* | 1. Digit recall  2. Counting recall  3. Dot matrix  4. Odd-one-out  (AWMA, 2007) | Cohen’s d | LOW |
| Engel et al. 2008 | Sao Paulo City, Brazil | | 40  (45%) | 6 – 7 years  (83.4±4.33) | 100% Brazilian | | Income, parental occupation, and parental education  [Composite]  *(low SEP n = 20, high SEP n = 20)* | 1. Counting recall  2. BDR  3. FDR  4. Psuedoword repetition | Cohen’s d | LOW |
| Finch and Obradović, 2017 | San Francisco Bay, USA | | 102  (48%) | 4 – 6 years  (5.61±0.56) | 36% White, 26% Hispanic/Latino, 20% Asian, 4% Black, 14% multiracial/other. | | Family income, parental education, subjective social status, financial stress  [Composite] | 1. BDR | Converted from Pearson’s r | LOW |
| Lawson and Farah, 2017 | USA | | 336  (48%) | 6 – 15 years  (10.13±2.94 at first time point] | 81% White, 8% African American/Black, 1% Asian, 2% Multiracial, 7% unknown ethnicity. | | (i) Family income  (ii) Parental education | 1. Corsi (CANTAB)  2. Spatial WM (CANTAB)  3. Digit span – FDR & BDR (WISC-III) | Converted from Pearson’s r | LOW |
| Lensing and Elsner, 2018 ** | Brandenburg, Germany | | 1596  (47%) | 6 – 7 years (7.35±0.41)  8 – 9 years  (8.90±0.52) | NR | | Maternal education | 1. BDR (WISC)  {at three time points} | Converted from Pearson’s r | LOW |
| Lima et al. 2020 | Brazil | | 569 (52%) | 7-12 years  (9.51±1.52) | NR | | (i) Maternal education  (ii) Family income | 1. Corsi Block Tapping Task – forwards 2. Corsi Block Tapping Task – backwards (Kessels et al. 2000) | Converted from Pearson’s r | LOW |
| Lipina et al. 2013 | Buenos Aires, Argentina | | 250  (46%) | NR  (4.87±0.59) | 100% Argentine | | Unsatisfied Basic Needs with at least one of: (1) inappropriate housing, (2) absence of waste systems in household, (3) overcrowding, (4) school-aged children not in education, (5) head of household with incomplete primary school  *(low SEP n = 92, high SEP n = 146)* | 1. Corsi blocks (Pickering, 2001) | Cohen’s d | HIGH |
| Madhushanthi et al., 2018 | Galle, Sri Lanka | | 200  (0%) | 11 – 14  (12.21±1.15) | 84% Sinhalese, 16% unknown | | (i) Maternal education  (ii) Parental education  (iii) Parental occupation  (iiii) Family income  [& composite for group analysis]  *(low SEP n = 112, high SEP n = 88)* | 1. Digit span (WISC-IV)  2. Arithmetic (WISC-IV)  3. Visuospatial (WISC-IV) | Cohen’s d | LOW |
| Malda et al. 2010 | South Africa | | 501  (51%) | Grades 3 - 4  (9.37±1.05) | 32% White Urban Afrikaans, 36% Black urban Tswana, 32% Black rural Tswana | | Children were asked six questions as an indication of SES: (1) do you have your own room? (£) how many TVs are there in your house (3) is there a microwave in your house? (4) how many cellphones does your family have? (5) how many cars does your family have? (6) do you have reading books at home?  [Composite] | 1. Short term memory  2. WM test (WMTB-C, 2001)  {adapted for two different cultures} | Converted from Pearson’s r | HIGH |
| Markovits and Brunet, 2012 | Montreal, Canada | | 205  (49%) | Grade 1 (6 years 4 months)  Grade 2 (7 years 5 months) | NR | | Lower: two low SEP public school in poor districts *(n = 92)*  Higher: one high SEP public school in suburbs of Montreal *(n = 113)* | 1. Digit span | Cohen’s d | HIGH |
| Metaferia et al. 2020 | Ethiopa | | 102 (56%) | 50-74 months  (62.08±7.66) | NR | | Parent education and income  [Composite] | 1. Mr Peanut task (Kemps et al., 2000; Morra, 1994) | Converted from Pearson’s r | HIGH |
| Ming et al. 2021 | Southern and Northern China | | 888 (57%) | 9-13 years (10.68±1.07) | NR | | Family income, parental education, parental occupation  [Composite] | 1. Visual patterns test (Sala et al, 1999) | Converted from Pearson’s r | HIGH |
| Nesbitt et al. 2013 ** | NR | | 206  (51%) | 6, 12, 30, and 36 months at 4 data collections | 43% European American, 57% African American | | (i) Income to needs  (ii) Maternal education  (Aggregated from 6 – 36 months visits) | 1. BDR (McCarthy Scales of Children’s Abilities, 1972)  (measured during kindergarten age) | Converted from Pearson’s r | LOW |
| Noble et al. 2007 | New York City | | 168  (48%) | First grade  (NR) | 34% African-American, 7% Asian, 23% Latino, 23% White, 14% Mixed/other | | Parental education, income-to-needs, parental occupation  [Composite] | 1. Spatial WM (Klingberg et al. 2002)  2. Delayed nonmatch to sample (Marks et al. 2001)  {composite and individual tests} | Converted from Pearson’s r | LOW |
| Noble et al. 2005 | Philidelphia | | 60  (50%) | NR  (5 years 10 months ±NR) | 100% African American | | Parental education, parental occupation, income-to-needs  *(Low SEP n = 30, high SEP n = 30)* | 1. Spatial WM (Hughes, 1998) | Cohen’s d | LOW |
| Philbrook et al. 2017** | Southeastern United States | | 282  (52%) | 9 – 11 years  (9.44±0.71, at the first wave) | 65% European American, 35% African American | | Income-to-needs ratio | 1. WM test (WJ-III) | Converted from Pearson’s r | LOW |
| Pina et al. 2014 ** | Mucia, Southeastern Spain | | 102  (45%) | 9 – 13 years  (10 years ± 11 months) | 74% Spanish speakers born in Spain, 8% born outside Spain by non Spanish parents, 18% unknown | | Parental education | 1. Corsi forward (Kessels et al 2001, 2008)  2. Corsi Backward (Kessels et al 2001, 2008)  3. FDR  4. BDR | Converted from Pearson’s r | LOW |
| Riva et al. 2017 | Italy | | 646  (female:male ratio = .9) | 6 – 11 years  (8.22±1.17) | 100% Italian | | Parental occupation (Hollingshead, 1975) | 1. FDR  2. BDR | Converted from Pearson’s r | LOW |
| Rosen et al. 2020** | Seattle, USA | | 101 (50%) | 60-75 months (5.55±0.37) | 67.3% White, 14.8% Black, 2.9% American Indian or Alaska Native, 12.8% Asian, 0.9% Native Hawaiian or Pacific Islander, 0.9% Other; 8.9% Hispanic or Latino | | (i) Income to needs  (ii) Parental education | 1. BDR | Converted from Pearson’s r | LOW |
| St John et al. 2019 | USA | | 121  (42%) | 4 – 5 years old  (5.02±0.29) | 43% White, 12% Black, 11% Hispanic, 10% Asian, 22% Multiracial | | Maternal and paternal education level, household income, household composition, and maternal and paternal occupation.  [Composite] | 1. Change detection task – accuracy at two set sizes  2. Change detection task – reaction time at two set sizes  (Luck and Vogel, 1997) | Converted from Pearson’s r | LOW |
| Stumper et al. 2020** | USA  Adolescent Cognition and Emotion (ACE) project | | 243 (47%) | 12-16  (13.01±.79)  (14.09±.81) | 47.3% White/Caucasian, 49.0% Black/African American, 3.7% Biracial/other  Individuals who identified as members of other racial or ethnic groups were excluded. | | (i) Family income  (ii) Maternal education | 1. FDR  2. BDR  (WISC-IV; Wechsler, 2003) | Converted from Pearson’s r | LOW |
| Suor et al. 2017 | Northeastern USA | | 185  (53%) | 3.5 – 5 years old  (NR) | 59% European American, 19% African American, 3% Latino, 15% Biracial, 1% Asian, 3% Native American/Alaskan | | (i) Maternal education at age 3.5  (ii) Income-to-needs  (iii) Neighbourhood characteristics  (iiii) Family SES composite | 1. Backword word span at age 5 (Carlson, Moses and Breton, 2005)  2. PathSpan application (Hume, 2012)  {Individual and composites}  {WM at age 5} | Converted from Pearson’s r | LOW |
| Wang and Fitzpatrick,. 2019** | USA  Early Childhood Longitudinal Study-Kindergarten class | | 14,000  (51%) | 4-8 years  (NR) | 50% White, non-Hispanic, 13% Black/African American, non-Hispanic, 24% Hispanic, 7% Asian, non-Hispanic, 1% Native Hawaiian/Pacific Islander, non-Hispanic, 1% American Indian/Alaska native, non-Hispanic, 4% Two or more races, non-Hispanic | | Family income and parental education  [Composite] | 1. BDR | Converted from Pearson’s r | LOW |
| Waters et al 2021 | USA  National Institute of Child Health and Development Study of Early Child Care and Youth Development (NICHD SECCYD) | | 990 (52%) | 4-5 years  (4.64±0.09) | 86% White, 14% Black | | (i) Income to needs  (ii) Parent education | 1. Memory for sentences  (Woodcock-Johnson Revised; Woodcock and Johnson, 1989) | Converted from Pearson’s r | LOW |
| Wei et al. 2020 | Shanghai, China | | 173  (51%) | (67.25±3.67 months) | NR, Native Mandarin speakers | | Parent education, parent monthly income  [Composite] | 1. BDR  (WISC-R; Wechsler, 1974) | Converted from Pearson’s r | LOW |
| Wiebe et al. 2008 | NR | | 243  (44) | 2 – 6 years old  (3 years 11 months±12 months) | 70% White, 18% African American, 4% Asian American, 0.4 Native American, 1.7% Hispanic, 6% Multiracial | | Maternal education in years | 1. Delayed alternation (Espy et al. 1999)  2. Six boxes  3. Digit span (Elliott, 1990) | Converted from Pearson’s r | LOW |
|  | | **Studies included in Harvest plot** | | | | | | | | |
| Aran-Filippetti & Richaud De Minzi, 2012 | Santa Fe, Argentina | | 254  (50%) | 7 – 12  (9.66±1.29) | 100% Argentinian | | Class of neighbourhoods and socioeconomic coefficient of schools  [Composite]  *(low SEP n = 129, high SEP n = 125)* | 1. Digit span  2. Letter-number sequencing  (WM Index of WISC-IV)  [Composite] | *P* value (stepwise regression) | LOW |
| Brito et al., 2021 | New York City, USA | | 92 (61%) | 18 months  (18.51±0.66) | NR, range of both monolingual and bilingual speakers | | Maternal education, income, and income to needs  [Composite] | 1. Hide the pots (Bernier, Carlson & Whipple, 2010) | *P value (ANOVA)* | LOW |
| Cockcroft, 2016 | South Africa | | 120  (51%) | 6 – 8 years  (6.73±.63) | 55% Monolingual English speakers, 45% bilingual English and African speakers | | Living Standards Measure (South African Audience Research Foundation, 2001 – number of people in household, type of dwelling, housing tenure, area). Also occupational status and highest educational level.  [Composite] | 1. Digit recall  2. Non-word recall  3. Counting recall  4. BDR  (AWMA, 2007) | *P* value  (two-way Mancova) | LOW |
| Daubert and Ramani, 2020 | Mid-Atlantic USA | | 74 (47%) | 4-5 years  (4.11±NR) | 58% Caucasian/White, 23% African American or Black, 11% Asian or Pacific Islander, 7% bi or multiracial, 1% American Indian or Alaska Native | | Parent education and household income  [Composite] | 1. Frog matrices task   (Morales et al., 2013) | *P* value  (Regression) | LOW |
| Dicataldo and Roch, 2020 | Padua, Northeast Italy | | 115 (54%) | 44-75 months  (61.9±6.8) | NR, range of both monolingual and bilingual speakers | | Parental education and annual family income  [Composite] | 1. FDR 2. BDR   (WISC)  [Composite] | *P* value (Pearson’s r correlation) | HIGH |
| Dilworth-Bart, 2012 | Mid-west USA | | 49  (53%) | 54 – 66 months  (NR) | 22% African American/Black, 2% Asian/Pacific islander, 61% White, 14% multi-racial | | Maternal education and household income  [Composite] | 1. Verbal WM  2. Nonverbal WM  [Composite]  (SB5; Roid, 2003) | *P* value (Pearson’s r correlation) | HIGH |
| Farah et al. 2006 | Philadelphia | | 60  (43%) | 10 – 13 years  (11.7±1.0) | 100% African American | | Parental occupation (Hollingshead), parental education, and low SEP mothers on state and medical assistance  [Composite] | 1. Spatial WM (CANTAB, 1997)  2. Two-back | *P* value (Mancova) | LOW |
| Fernald et al., 2011 | Madagascar | | 1232 (48%) | 3 – 6  (NR) | NR | | Maternal education and household wealth  [Composite]  *(groups of maternal education n: none = 286, primary = 692, secondary and above = 254)* | 1. WM subtest (SB5)  2. Memory of phrases (Woodcock and Munoz, 1996) | *P* value (linear regression) | HIGH |
| Flouri et al., 2019** | UK  Millennium Cohort Study (MCS) | | 4756 | 0 – 11 years | 74% White, 26% NR | | (i) Maternal education  (ii) Family poverty (household income below poverty line)  (iii) Neighbourhood deprivation | 1. Spatial working memory at age 11 (CANTAB; Robbins et al., 1994) | *P* value  (multilevel regression model) | LOW |
| Guerra et al. 2020 | Rio Grande do Norte, Brazil | | 230 | 7-12 years | 100% Brazillian | | Public and private schools  (low SEP n = 116, high SEP n = 114) | 1. Visuospatial updating 2. Verbal updating   (Child Executive Function Battery; Roy et al., 2020) | *P* value (ANOVA) | LOW |
| Hou et al. 2020 | Anhui, China | | 142 | 9-10 years (10.01±2.62) | NR | | (i) Paternal education  (ii) Maternal education  (iii) Monthly family income | 1. FDR and BDR  2. Letter number sequencing  (WISC-IV) | *P value (correlations)* | LOW |
| Hackman et al. 2014 ** | NR | | 316  (46%) | 10 – 13 years  & four years later  (14.05±0.9) | 61% White, 26% African American, 10% Asian/Pacific Islander, .3% Native American, 3% Mixed, 8% Hispanic/Latino | | (i) Parental education  (ii) neighbourhood disadvantage | 1. BDR (WISC-IV)  2. Corsi  3. Spatial WM  4. Object two-back  [Composite] | *P* value (Multilevel model) | LOW |
| Hackman et al. 2015 ** | USA | | 1009  (50%) | 1 – 54 months  (NR) | 83% White, 11% African-American, 1% Asian/Pacific Islander, 0.2% American Indian, 4% Other, 5% Hispanic/Latino. | | (i) Income to needs average from 1, 6, 15, 24, and 26 months  (ii) Maternal education at 1 month | 1. Memory for sentences (WJ-R COG; Woodcock, 1990) [measured at 54 months] | *P* value (Multilevel model) | LOW |
| He and Yin, 2016 | Shaanxi, China | | 157  (59%) | 8 – 12 years  (9.9±1.31) | 100% Chinese | | Subjective family material environment (Adler et al. 2010)  Parental education and occupation (Hollingshead)  [Composite] | 1. FDR  2. BDR  (WISC-III)  [Composite] | *P* value (Partial correlations) | LOW |
| Jacobsen et al. 2017 | Porto Alegre, Brazil | | 274  (45%) | 6 – 12 years  (8.92±1.90) | NR | | Brazilian Economic Classification (parental education and living conditions) | Random Number Generation (Towse and Neil, 1998) | *P* value (Linear regression) | HIGH |
| Kobrosly et al. 2011 ** | Seychelles | | 463  (48%) | 6 months – 17 years  (NR) | 100% Seychellois | | (i) Hollingshead Social Status Index (maternal occupation and education) at 6 months  (ii) 107 months  (iii) 17 years | 1. Delayed match to sample  2. Spatial recognition memory  3. Spatial WM  (CANTAB)  {at 17 years old} | *P* value (Linear regression) | LOW |
| Korecky-Kroll et al., 2019 | Vienna | | 56 (50%) | 49 – 56 months (53.12±1.4) | 52% monolingual German speaking, 48% bilingual Turkish and German speaking | | Parental education and parental occupation  [Composite] | 1. Phonological working memory (SETK 3-5; Grimm, 2001) | *P* value (Kruskal Wallis test) | LOW |
| Leonard et al. 2015 | USA | | 58  (47%) | NR  (14.41±0.42) | Lower-SES group: 22% African American, 4% Asian, 54% White, 4% Native Hawaiian or Pacific Islander, 26% multiple races, 35% not Hispanic, 65% Hispanic, 35% did not report race.  Higher-SES group: 6% African American, 14% Asian, 54% White, 3% Native Hawaiian or Pacific Islander, 17% multiple races, 6% did not report race; 91% not Hispanic, 3% Hispanic, 6% did not report ethnicity | | Free or reduced school meals  *(low SEP n = 23, high SEP n = 35)* | 1. Counting span (Conway et al. 2005, Cowan et al. 2005) | *P* value (Anova) | LOW |
| Maguire and Schneider, 2019 | NR | | 90 (40%) | 8-15 years (10.9±2.14) | NR, 100% fluent English speakers and 37% Spanish-English bilingual speakers | | Maternal education | 1. Digit span (Blackburn & Benton, 1957)  (NR if forwards or backwards) | *P* value (Linear regression) | HIGH |
| Miconi et al. 2019 | Northeast Italy | | 488  (NR but balanced) | 11 – 13 years  (12.11±0.80) | 24% Moroccan immigrants, 25% Romanian immigrants, 51% non-immigrant Italians | | Family Affluence Scale ( Currie et al., 2008)  Material affluence reported by adolescents themselves | 1. FDR  2. BDR  [Composite] | *P* value (Bivariate correlations) | LOW |
| Murtaza et al. 2019 | Negeri Sembilan, Malaysia | | 269 (51%) | 2-6 years (4.03±1.21) | Indigenous Orang Asli | | (i) Maternal education  (ii) Paternal education  (iii) Maternal income  (iiii) Paternal income | 1. Picture memory  2. Zoo location  (WMI, WPPSY-IV)  [Composite] | *P* value (Linear regression) | LOW |
| Passareli-Carrazzoni et al. 2018 | Sao Paulo state, Brazil | | 96  (52%) | 9 – 10 years  (9.5±0.5) | NR | | (i) Family composition (one or two parents)  (ii) Monthly family income  (iii) Maternal schooling in years | 1. Digit span  2. Arithmetic  3. Letter-number-sequencing  [Composite] | *P* value (Linear regression) | LOW |
| Piccolo et al. 2019 | USA | | 108  (58%) | 9 – 18 years  (14.10±1.76) | NR | | (i) Yearly family income  (ii) Parental educational attainment | 1. List-sorting WM test (NR) | *P* value (Linear regression) | HIGH |
| Rhoades, 2012 ** | Pennsylvania | | 1155  (approx. 50%) | 2, 7, 24, and 36 months old at each visit | 60% White, 40% African American | | LCA to create risk classes in different ethnic groups based on household income, unmarried, partner status, teen mother, no high school diploma, mood problems, smking during pregnancy, high stress, low social support, and crowded house | 1. WM task (NR) | *P* value (Linear regression) | LOW |
| Rowe et al. 2016 | California | | 501  (48%) | 6 month, 1, 2, 3.5, 5, 7, 9 and 10.5 years old at each visit | 96% Latina, 4% other ethnicity | | (i) Household poverty status as a binary variable  (ii) Neighbourhood poverty status in quartiles)  (At the 10.5 year visit) | 1. WM subscale (WISC-IV)  (at 10.5 year visit) | *P* value (Linear regression) | LOW |
| Sarsour et al. 2011 | San-Francisco | | 60  (31%) | 8 – 12 years  (9.9±0.96) | 100% Brazilian | | The MacArthur Research Network on SES and Health questionnaire, family income-to0needs ratio, parental occupation via Hollingshead (1975), family wealth, and maternal education  [Composite] | 1. Pseudo word span  2. FDR  3. BDR  [Composite]  (Child Brief Neuropsychological Assessment Battery, Salles et al. 2011) | *P* value (Correlation) | LOW |
| Tine, 2014 | NR | | 186  (52%) | 10 – 12 years  (11.3±NR) | In rural schools; 96% White.  Low-income urban schools: 62% ethnic minority.  High income urban school: 36% ethnic minority  (Ethnic minorities include American Indian, Alaskan Native, Asian, Black/African American, or Pacific Islander) | | Low SEP: 1) Attended a school that serves a community with a median family income below the national median family income of $50,033. 2) Attended a school in which at least 75% of students qualify for FSM or reduced. 3) They themselves qualified for FSM. Divided into urban and rural schools *(n = 94)*  High SEP: 1) Attended a school that serves a community with a median family income above the national median. 2) Less than 25% of the school were on FSM. 3) They did not qualify for FSM. Divided into urban and rural schools *(n = 92)* | 1. Listening recall  2. BDR  3. Odd-one-out  4. Mr X  (AWMA, 2007) | *P* value (t-test) | LOW |
| Vandenbroucke et al. 2016 | Belgium | | 78  (65%) | 5 – 6 years old  (5.88±0.29) | 92.5% Monolingual Dutch speakers born in Belgium, 8% Bilingual, 5% not Belgium born | | Low SEP: single parent and low-income families, with a young low-educated mother who more often smoked during pregnancy *(n = 21)*  High SEP: mainly two-biological-parent high income families, with a highly educated mother who did not smoke during pregnancy *(n = 57)* | 1. Verbal WM: Digit recall, word recall, listening recall, BDR  2. Visuospatial WM: dot matrix, block recall, odd-one-out and Mr-X  (AWMA; Alloway, 2007) | *P* value (t-test) | LOW |

Note regarding SEP indicator: Individual SEP indicators are listed as (i), (ii), etc, and composite SEP indicators are indicated as such with square brackets. SEP indicators are assumed continuous, unless *n* is specified for high and low SEP groups.

Abbreviations: Backwards Digit Recall (BDR), Forwards Digit Recall (FDR), Working Memory Test Battery for Children (WMTB-C), Wechsler Intelligence Scales for Children (WISC), Automated Working Memory Assessment (AWMA), Woodcock Johnson (WJ), Stanford Binet Intelligence Scales for Early Childhood, 5th edition (SB5), Cambridge Neuropsychological Test Automated Battery (CANTAB) and not reported (NR).
