# Supplementary material for: The association between socioeconomic disadvantage and children’s working memory abilities: A systematic review and meta-analysis
Source: PLoS One. 2021 Dec 2;16(12):e0260788. doi: 10.1371/journal.pone.0260788 (PMC8639069; doi:10.1371/journal.pone.0260788)
Supplement: S3 File — (DOCX) [file pone.0260788.s003.docx]

# Supplementary online materials

## 3. References for studies included in systematic review

1. Albert WD, Hanson JL, Skinner AT, Dodge KA, Steinberg L, Deater-Deckard K, et al. Individual differences in executive function partially explain the socioeconomic gradient in middle-school academic achievement. Dev Sci [Internet]. 2020;23(5):e12937. Available from: http://ovidsp.ovid.com/ovidweb.cgi?T=JS&PAGE=reference&D=med17&NEWS=N&AN=31912610
2. Alloway TP, Alloway RG, Wootan S. Home sweet home: Does where you live matter to working memory and other cognitive skills? J Exp Child Psychol [Internet]. 2014;124(1):124–31. Available from: http://dx.doi.org/10.1016/j.jecp.2013.11.012
3. Arán-Filippetti V. Structure and invariance of executive functioning tasks across socioeconomic status: Evidence from Spanish-speaking children. Span J Psychol. 2013;16(January 2013).
4. Arán-Filippetti V, Richaud De Minzi MC. A structural analysis of executive functions and socioeconomic status in school-age children: Cognitive factors as effect mediators. J Genet Psychol [Internet]. 2012 Oct 1 [cited 2020 Nov 17];173(4):393–416. Available from: https://www.tandfonline.com/doi/abs/10.1080/00221325.2011.602374
5. Babayiǧit S. Contributions of word-level and verbal skills to written expression: Comparison of learners who speak English as a first (L1) and second language (L2). Read Writ [Internet]. 2014 Oct 31 [cited 2020 Nov 17];27(7):1207–29. Available from: https://link.springer.com/article/10.1007/s11145-013-9482-z
6. Bowey JA. Socioeconomic status differences in preschool phonological sensitivity and first-grade reading achievement. Journal of Educational Psychology; 1995.
7. Brito NH, Greaves A, Leon-Santos A, Fifer WP, Noble KG. Associations between bilingualism and memory generalization during infancy: Does socioeconomic status matter? Biling Lang Cogn [Internet]. 2021;24(2):231–40. Available from: http://ovidsp.ovid.com/ovidweb.cgi?T=JS&PAGE=reference&D=psyc17&NEWS=N&AN=2020-36761-001
8. Carlson SM, Meltzoff AN. Bilingual experience and executive functioning in young children. Dev Sci [Internet]. 2008 Mar 1 [cited 2020 Nov 17];11(2):282–98. Available from: https://onlinelibrary.wiley.com/doi/full/10.1111/j.1467-7687.2008.00675.x
9. Catale C, Willems S, Lejeune C, Meulemans T. Parental educational level influence on memory and executive performance in children. Rev Eur Psychol Appl [Internet]. 2012;62(3):161–71. Available from: http://dx.doi.org/10.1016/j.erap.2012.04.003
10. Chung KKH, Liu H, McBride C, Wong AM-Y, Lo JCM. How socioeconomic status, executive functioning and verbal interactions contribute to early academic achievement in Chinese children. Educ Psychol [Internet]. 2017 Apr 21 [cited 2020 Nov 17];37(4):402–20. Available from: https://www.tandfonline.com/doi/full/10.1080/01443410.2016.1179264
11. Cockcroft K. A comparison between verbal working memory and vocabulary in bilingual and monolingual South African school beginners: Implications for bilingual language assessment. Int J Biling Educ Biling [Internet]. 2016;19(1):74–88. Available from: http://dx.doi.org/10.1080/13670050.2014.964172
12. Corso HV, Cromley JG, Sperb T, Salles JF. Modeling the relationship among reading comprehension, intelligence, socioeconomic status, and neuropsychological functions: The mediating role of executive functions. Psychol Neurosci [Internet]. 2016 Mar 1 [cited 2020 Nov 17];9(1):32–45. Available from: /record/2016-05583-001
13. Daubert EN, Ramani GB. Math and Memory in Bilingual Preschoolers: The Relations Between Bilingualism, Working Memory, and Numerical Knowledge. J Cogn Dev. 2019 May 27;20(3):314–33.
14. Deer LK, Hastings PD, Hostinar CE. The Role of Childhood Executive Function in Explaining Income Disparities in Long-Term Academic Achievement. Child Dev [Internet]. 2020;91(5):e1046–63. Available from: http://ovidsp.ovid.com/ovidweb.cgi?T=JS&PAGE=reference&D=prem&NEWS=N&AN=32712955
15. Dicataldo R, Roch M. Are the effects of variation in quantity of daily bilingual exposure and socioeconomic status on language and cognitive abilities independent in preschool children? Int J Environ Res Public Health [Internet]. 2020;17(12):1–23. Available from: https://www.mdpi.com/1660-4601/17/12/4569/pdf
16. Dilworth-Bart JE. Does executive function mediate SES and home quality associations with academic readiness? Early Child Res Q. 2012;27(3):416–25.
17. Engel de Abreu PMJ, Nikaedo C, Abreu N, Tourinho CJ, Miranda MC, Bueno OFA, et al. Working Memory Screening, School Context, and Socioeconomic Status: An Analysis of the Effectiveness of the Working Memory Rating Scale in Brazil. J Atten Disord [Internet]. 2014 May 8 [cited 2020 Nov 17];18(4):346–56. Available from: http://journals.sagepub.com/doi/10.1177/1087054713476138
18. Engel PMJ, Santos FH, Gathercole SE. Are working memory measures free of socio-economic influence? Anim Genet. 2008;39(5):561–3.
19. Farah MJ, Shera DM, Savage JH, Betancourt L, Giannetta JM, Brodsky NL, et al. Childhood poverty: Specific associations with neurocognitive development. Brain Res. 2006;1110(1):166–74.
20. Fernald LCH, Weber A, Galasso E, Ratsifandrihamanana L. Socioeconomic gradients and child development in a very low income population: Evidence from Madagascar. Dev Sci [Internet]. 2011 Jul 1 [cited 2020 Nov 17];14(4):832–47. Available from: https://onlinelibrary.wiley.com/doi/full/10.1111/j.1467-7687.2010.01032.x
21. Finch JE, Obradović J. Unique effects of socioeconomic and emotional parental challenges on children’s executive functions. J Appl Dev Psychol. 2017;52(October 2016):126–37.
22. Flouri E, Papachristou E, Midouhas E. The role of neighbourhood greenspace in children’s spatial working memory. Br J Educ Psychol [Internet]. 2019;89(2):359–73. Available from: http://ovidsp.ovid.com/ovidweb.cgi?T=JS&PAGE=reference&D=emed20&NEWS=N&AN=627820732
23. Guerra A, Hazin I, Guerra Y, Roulin J-L, Le Gall D, Roy A. Developmental Profile of Executive Functioning in School-Age Children From Northeast Brazil. Front Psychol [Internet]. 2020;11:596075. Available from: http://ovidsp.ovid.com/ovidweb.cgi?T=JS&PAGE=reference&D=pmnm&NEWS=N&AN=33536970
24. Hackman DA, Betancourt LM, Gallop R, Romer D, Brodsky NL, Hurt H, et al. Mapping the trajectory of socioeconomic disparity in working memory: Parental and neighborhood factors. Child Dev. 2014;85(4):1433–45.
25. Hackman DA, Gallop R, Evans GW, Farah MJ. Socioeconomic status and executive function: developmental trajectories and mediation. Dev Sci [Internet]. 2015 Sep 1 [cited 2018 Nov 2];18(5):686–702. Available from: http://doi.wiley.com/10.1111/desc.12246
26. He ZH, Yin WG. Family Environments and Children’s Executive Function: The Mediating Role of Children’s Affective State and Stress. J Genet Psychol [Internet]. 2016;177(5):143–55. Available from: http://dx.doi.org/10.1080/00221325.2016.1218322
27. Hou W-P, Tan TX, Wen Y-J, Wang X-Q, Li X-B, Wang C-Y. The effect of increased family finance and dual-parental absence since infancy on Children’s cognitive Abilities. Soc Sci Med [Internet]. 2020;266:113361. Available from: http://ovidsp.ovid.com/ovidweb.cgi?T=JS&PAGE=reference&D=medl&NEWS=N&AN=32978007
28. Jacobsen GM, de Mello CM, Kochhann R, Fonseca RP. Executive Functions in School-age Children: Influence of Age, Gender, School Type and Parental Education. Appl Cogn Psychol. 2017;31(4):404–13.
29. Kobrosly RW, Van Wijngaarden E, Galea S, Cory-Slechta DA, Love T, Hong C, et al. Socioeconomic position and cognitive function in the Seychelles: A life course analysis. Neuroepidemiology. 2011;36(3):162–8.
30. Korecky-Kroll K, Dobek N, Blaschitz V, Sommer-Lolei S, Boniecki M, Uzunkaya-Sharma K, et al. Vocabulary as a central link between phonological working memory and narrative competence: Evidence from monolingual and bilingual four-year-olds from different socioeconomic backgrounds. Lang Speech [Internet]. 2019;62(3):546–69. Available from: http://ovidsp.ovid.com/ovidweb.cgi?T=JS&PAGE=reference&D=psyc17&NEWS=N&AN=2019-48657-007
31. Lawson GM, Farah MJ. Executive function as a mediator between SES and academic achievement throughout childhood. Int J Behav Dev. 2017;41(1):94–104.
32. Lensing N, Elsner B. Development of hot and cool executive functions in middle childhood: Three-year growth curves of decision making and working memory updating. J Exp Child Psychol. 2018 Sep 1;173:187–204.
33. Leonard JA, Mackey AP, Finn AS, Gabrieli JDE. Differential effects of socioeconomic status on working and procedural memory systems. Front Hum Neurosci [Internet]. 2015 Oct 8 [cited 2020 Nov 17];9(OCT):554. Available from: http://journal.frontiersin.org/Article/10.3389/fnhum.2015.00554/abstract
34. Lima CS, Souza Marques B, Ferreira Carvalho C, Siquara GM, Bezerra MLO, Duarte TS, et al. Visuospatial working memory: A socioeconomic normative reference of the Corsi Block-Tapping Task for children aged 7 to 12 years old in Brazil. Psychol Neurosci [Internet]. 2020;13(4):503–15. Available from: http://ovidsp.ovid.com/ovidweb.cgi?T=JS&PAGE=reference&D=psyc17&NEWS=N&AN=2020-20022-001
35. Lipina S, Segretin S, Hermida J, Prats L, Fracchia C, Camelo JL, et al. Linking childhood poverty and cognition: Environmental mediators of non-verbal executive control in an Argentine sample. Dev Sci. 2013;16(5):697–707.
36. Madhushanthi HJ, Wimalasekera SW, Goonewardena CSE, Amarasekara AATD, Lenora J. Socioeconomic status is a predictor of neurocognitive performance of early female adolescents. Int J Adolesc Med Health. 2018;(June).
37. Maguire MJ, Schneider JM. Socioeconomic status related differences in resting state EEG activity correspond to differences in vocabulary and working memory in grade school. Brain Cogn [Internet]. 2019;137:103619. Available from: http://ovidsp.ovid.com/ovidweb.cgi?T=JS&PAGE=reference&D=med16&NEWS=N&AN=31655309
38. Malda M, van de Vijver FJR, Temane QM. Rugby versus Soccer in South Africa: Content familiarity contributes to cross-cultural differences in cognitive test scores. Intelligence [Internet]. 2010;38(6):582–95. Available from: http://dx.doi.org/10.1016/j.intell.2010.07.004
39. Markovits H, Brunet M-L. Priming divergent thinking promotes logical reasoning in 6- to 8-year olds: But more for high than low SES students. J Cogn Psychol [Internet]. 2012 Dec 1 [cited 2020 Nov 17];24(8):991–1001. Available from: http://www.tandfonline.com/doi/abs/10.1080/20445911.2012.729034
40. Metaferia BK, Takacs ZK, Futo J. The Relationship Between Parental Play Beliefs, Preschoolers’ Home Experience, and Executive Functions: An Exploratory Study in Ethiopia. Front Psychol [Internet]. 2020;11:624. Available from: http://ovidsp.ovid.com/ovidweb.cgi?T=JS&PAGE=reference&D=pmnm&NEWS=N&AN=32373015
41. Miconi D, Moscardino U, Altoè G, Salcuni S. Self‐Construals and Social Adjustment in Immigrant and Nonimmigrant Early Adolescents: The Moderating Role of Executive Functioning. Child Dev [Internet]. 2019 Jan 21 [cited 2020 Nov 17];90(1):e37–55. Available from: https://onlinelibrary.wiley.com/doi/10.1111/cdev.12918
42. Ming H, Zhang F, Jiang Y, Ren Y, Huang S. Family socio-economic status and children’s executive function: The moderating effects of parental subjective socio-economic status and children’s subjective social mobility. Br J Psychol [Internet]. 2021; Available from: http://ovidsp.ovid.com/ovidweb.cgi?T=JS&PAGE=reference&D=emexb&NEWS=N&AN=633975565
43. Murtaza SF, Gan WY, Sulaiman N, Shariff ZM, Ismail SIF. Sociodemographic, nutritional, and environmental factors are associated with cognitive performance among Orang Asli children in Malaysia. PLoS One [Internet]. 2019;14(7). Available from: http://ovidsp.ovid.com/ovidweb.cgi?T=JS&PAGE=reference&D=psyc17&NEWS=N&AN=2019-41143-001
44. Nesbitt KT, Baker-Ward L, Willoughby MT. Executive function mediates socio-economic and racial differences in early academic achievement. Early Child Res Q [Internet]. 2013;28(4):774–83. Available from: http://dx.doi.org/10.1016/j.ecresq.2013.07.005
45. Noble KG, McCandliss BD, Farah MJ. Socioeconomic gradients predict individual differences in neurocognitive abilities. Dev Sci. 2007;10(4):464–80.
46. Noble KG, Norman MF, Farah MJ. Neurocognitive correlates of socioeconomic status in kindergarten children. Dev Sci [Internet]. 2005 Jan 1 [cited 2018 Oct 22];8(1):74–87. Available from: http://doi.wiley.com/10.1111/j.1467-7687.2005.00394.x
47. Passareli-Carrazzoni P, Pereira-Lima K, Loureiro SR. Children’s working memory: Maternal and child sociodemographic, cognitive, and mental health predictors. Psychol Neurosci [Internet]. 2018 Jun 1 [cited 2020 Nov 17];11(2):146–54. Available from: /record/2018-28694-003
48. Philbrook, L E. Sleep and Cognitive Functioning in Childhood: Ethnicity, Socioeconomic Status, and Sex as Moderators. Physiol Behav. 2017;176(12):139–48.
49. Piccolo LR, Merz EC, Noble KG. School climate is associated with cortical thickness and executive function in children and adolescents. Dev Sci [Internet]. 2019 Jan 29 [cited 2020 Nov 17];22(1):e12719. Available from: https://onlinelibrary.wiley.com/doi/abs/10.1111/desc.12719
50. Pina V, Fuentes LJ, Castillo A, Diamantopoulou S. Disentangling the effects of working memory, language, parental education, and non-verbal intelligence on children’s mathematical abilities. Front Psychol. 2014;5(MAY):1–12.
51. Rhoades BL, Greenberg MT, Lanza ST, Blair C. Demographic and familial predictors of early executive function development: Contribution of a person-centered perspective. J Exp Child Psychol [Internet]. 2011;108(3):638–62. Available from: http://dx.doi.org/10.1016/j.jecp.2010.08.004
52. Riva V, Cantiani C, Dionne G, Marini A, Mascheretti S, Molteni M. Working memory mediates the effects of gestational age at birth on expressive language development in children. Neuropsychology [Internet]. 2017 Jul 1 [cited 2020 Nov 17];31(5):475–85. Available from: /record/2017-15236-001
53. Rosen ML, Hagen MP, Lurie LA, Miles ZE, Sheridan MA, Meltzoff AN, et al. Cognitive Stimulation as a Mechanism Linking Socioeconomic Status With Executive Function: A Longitudinal Investigation. Child Dev [Internet]. 2020;91(4):e762–79. Available from: http://ovidsp.ovid.com/ovidweb.cgi?T=JS&PAGE=reference&D=medl&NEWS=N&AN=31591711
54. Rowe C, Gunier R, Bradman A, Harley KG, Kogut K, Parra K, et al. Residential proximity to organophosphate and carbamate pesticide use during pregnancy, poverty during childhood, and cognitive functioning in 10-year-old children. Environ Res. 2016 Oct 1;150:128–37.
55. Sarsour K, Sheridan M, Jutte D, Nuru-Jeter A, Hinshaw S, Boyce WT. Family socioeconomic status and child executive functions: The roles of language, home environment, and single parenthood. J Int Neuropsychol Soc. 2011;17(1):120–32.
56. St. John AM, Kibbe M, Tarullo AR. A systematic assessment of socioeconomic status and executive functioning in early childhood. J Exp Child Psychol [Internet]. 2018;178:352–68. Available from: https://doi.org/10.1016/j.jecp.2018.09.003
57. Stumper A, Mac Giollabhui N, Abramson LY, Alloy LB. Early Pubertal Timing Mediates the Association between Low Socioeconomic Status and Poor Attention and Executive Functioning in a Diverse Community Sample of Adolescents. J Youth Adolesc [Internet]. 2020;49(7):1420–32. Available from: http://ovidsp.ovid.com/ovidweb.cgi?T=JS&PAGE=reference&D=med17&NEWS=N&AN=32020488
58. Suor JH, Sturge-Apple ML, Skibo MA. Breaking cycles of risk: The mitigating role of maternal working memory in associations among socioeconomic status, early caregiving, and children’s working memory. Dev Psychopathol. 2017 Oct 1;29(4):1133–47.
59. Tine M. Working Memory Differences Between Children Living in Rural and Urban Poverty. J Cogn Dev [Internet]. 2014 Oct 2 [cited 2020 Nov 17];15(4):599–613. Available from: http://www.tandfonline.com/doi/abs/10.1080/15248372.2013.797906
60. Vandenbroucke L, Verschueren K, Ceulemans E, De Smedt B, De Roover K, Baeyens D. Family demographic profiles and their relationship with the quality of executive functioning subcomponents in kindergarten. Br J Dev Psychol. 2016;34(2):226–44.
61. Wang AH, Fitzpatrick C. Which Early Childhood Experiences and Skills Predict Kindergarten Working Memory? J Dev Behav Pediatr. 2019;40(1):40–8.
62. Waters NE, Ahmed SF, Tang S, Morrison FJ, Davis-Kean PE. Pathways from Socioeconomic Status to Early Academic Achievement: The Role of Specific Executive Functions. Early Child Res Q [Internet]. 2021;54:321–31. Available from: http://ovidsp.ovid.com/ovidweb.cgi?T=JS&PAGE=reference&D=pmnm&NEWS=N&AN=33519062
63. Wei W, Li Y, Su H-Y. Predicting the growth patterns in early mathematics achievement from cognitive and environmental factors among Chinese kindergarten children. Learn Individ Differ [Internet]. 2020;79. Available from: http://ovidsp.ovid.com/ovidweb.cgi?T=JS&PAGE=reference&D=psyc17&NEWS=N&AN=2020-23296-001
64. Wiebe SA, Espy KA, Charak D. Using Confirmatory Factor Analysis to Understand Executive Control in Preschool Children: I. Latent Structure. Dev Psychol. 2008;44(2):575–87.
